# Changes in prevalence and patterns of consanguinity in Bradford, UK  – evidence from two cohort studies

**DOI:** 10.12688/wellcomeopenres.21121.2

**Published:** 2024-11-28

**Authors:** Neil Small, Brian Kelly, John Wright

**Affiliations:** 1 Faculty of Health Studies, University of Bradford, Bradford, England, UK; 2 Bradford Institute for Health Research, Bradford Teaching Hospitals NHS Foundation Trust, Bradford, England, UK

**Keywords:** cohort studies, consanguinity, congenital anomalies, Pakistani heritage

## Abstract

**Background:**

Research undertaken using the Born in Bradford cohort study identified consanguinity as a major risk factor for congenital anomalies and also reported longer term adverse health outcomes associated with consanguinity.

**Methods:**

We report the prevalence of consanguinity from two cohort studies in the same geographical area with a nine year gap: Born in Bradford (BiB) and Born in Bradford’s Better Start (BiBBS). We examine and compare rates of consanguinity and the characteristics of the consanguineous in each study population to examine if and how these have changed in the years between the recruitment periods of 2007–2010 (BiB) and 2016–2019 (BiBBS).

**Results:**

There had been a substantial decrease in consanguineous unions in women of Pakistani heritage, the proportion of women who were first cousins with the father of their baby fell from 39.3% to 27.0%, and those who were other blood relations fell from 23.1% to 19.3%. Only 37.6% of Pakistani heritage women were unrelated to the father of their baby in BiB, but 53.7% were unrelated in BiBBS. All but one White British respondent was unrelated to their baby’s father in both cohorts, and around 90% of the ‘Other ethnicities’ group (i.e., not White British or Pakistani heritage) were unrelated to the baby’s father in both cohorts. The reduction was most marked in women of Pakistani heritage who were born in the UK, in those educated to A level or higher and in women under age 25.

**Conclusions:**

An appreciation of changing rates of consanguinity and linked health needs will be valuable to those who commission and provide antenatal, paediatric and genetic services in Bradford and in other areas where consanguinity is likely to be a major risk factor. Falling rates in this city may reflect wider changes in partner choices in similar populations.

## Plain language summary

High rates of consanguinity (unions between blood relations, most often cousins) are a major risk factor for a particular category of genetic disorders called recessive disorders. These disorders can have a severe impact on children. There are also other longer term adverse health effects observed in children and adults from these unions. We report results from two ongoing cohort studies from the same city, Born in Bradford (BiB) and Born in Bradford’s Better Start (BiBBS). We examine and compare rates of consanguinity and the characteristics of the consanguineous in each study population to examine if and how these have changed in the years between the two cohort recruitment periods of 2007–2010 (BiB) and 2016–2019 (BiBBS).

In both cohorts the majority of women were of Pakistani heritage, 65% and 62% in the BiB and BiBBS respectively. Over a nine year period there had been a reduction in consanguineous unions in the Pakistani heritage cohort members. Only 38% of Pakistani heritage women were unrelated to the father of their baby in the BiB cohort, but 54% were unrelated in the BiBBS cohort. Rates of consanguinity in White British women and women in the “other ethnicities” group were very low in both cohorts. The reduction was most marked in women of Pakistani heritage who were born in the UK, in those educated to A level or higher and in women under age 25.

Comparing cohorts across time is a valuable way to examine social and behavioural change especially where the characteristics of the cohort members are similar. A reduction in consanguinity is likely to reduce the number of recessive genetic disorders and other illnesses linked to consanguinity.

## Introduction: Study background and context

A consanguineous union is one in which the male-female couple are related as second cousins or closer. One billion people, one in eight of the world’s population, live in countries where these are common (defined as rates of above 20% – see Bittles, 2012). Frequency of recessive genetic disorders among children born to consanguineous parents is around twice that of children of non-related parents ( Sheridan *et al.*, 2013) and there is evidence of increased rates of mortality and morbidity in these children ( Bishop *et al.*, 2018; Clark *et al.*, 2019; Lodh *et al.*, 2023; Malawsky *et al.*, 2023; Small *et al.*, 2024a).

Congenital anomalies occur in all communities. Rates are higher in children born to consanguineous parents although even here the considerable majority of children will not have an anomaly. In the BiB study about 3 in every 100 children born to non-consanguineous parents and about 6 in every 100 born to consanguineous partners were born with an anomaly. There are also other significant factors associated with increased presence of congenital anomalies. BiB reported an increase in risk of similar magnitude to consanguinity for non-consanguineous mothers of white British origin older than 34 years. Further, anomalies of different sorts and with risk factors other than consanguinity occur in all babies ( Sheridan *et al.,* 2013). But even though congenital anomalies occur in only a small proportion of children, the consequences of being born with one can be severe ( Small *et al.*, 2024a). These consequences include the death of the child or long-term morbidity. Given that there are high numbers of consanguineous parents in Bradford, and despite most of their children not having a congenital anomaly, the numbers dying or living with the consequences of a congenital anomaly in Bradford are significant to the city and to its health services.

We report the prevalence of consanguinity and the characteristics of the consanguineous from two ongoing UK birth cohort studies in Bradford, a city in the north of England, with a nine year gap: Born in Bradford (BiB), recruited between 2007 and 2010 ( Wright *et al.*, 2013), and Born in Bradford’s Better Start (BiBBS), recruited between 2016 and 2019 ( Dickerson *et al.*, 2022).

## Methods

### Data

Between 12 ^th^ March 2007 and 24 ^th^ December 2010 BiB collected detailed information from 12453 women with 13776 pregnancies and from 3448 of their partners ( Raynor & The Born in Bradford Collaborative Group, 2008; Wright *et al.*, 2013). A second cohort, BiBBS, was recruited between 4 ^th^ January 2016 and 30 ^th^ November 2019. It was made up of pregnant women living in three inner city wards, Bowling and Barkerend, Bradford Moor and Little Horton, all of which are within the wider BiB recruitment area ( Dickerson *et al.*, 2016; Dickerson *et al.*, 2022). There were 2392 women recruited with 2626 pregnancies in the BiBBS cohort; 2494 women in BiB were in the Better Start area. (See Small *et al.*, 2024b for a comparison of the characteristics of the total BiB cohort compared with the BiB women in the Better Start Area.) Data is at the pregnancy level, and some women had more than one pregnancy during each recruitment period. The relationship with the father of the baby could change between pregnancies for woman with more than one pregnancy. Also, a small number of women are in both cohorts including 237 women of Pakistani heritage.

In both cohorts the consanguinity status of women was captured in a self-administered questionnaire (see Small *et al.*, 2024b for relevant sections of each questionnaire). Women were classified as not related, first cousin, or other blood relation with the father of their child. Not all gave full answers to these questions and we exclude these from the subsequent analysis. The resulting figure in each cohort sees 2494 women in the BiB cohort in the Better Start area and 2564 in BiBBS. These constitute the sample used in this paper.

Other measures captured in both cohorts’ recruitment questionnaires and used in this analysis were ethnicity, age of woman at pregnancy, country of birth and age that women born outside the UK moved to the UK, self-reported financial status, and education. Ethnicity was grouped into three categories: White British, Pakistani heritage, and other ethnicity. Self-reported financial status was captured using a five-point Likert scale. Due to small numbers, we grouped the self-reported financial status categories of ‘finding it quite difficult’ and ‘finding it very difficult’ into a single category in this analysis. The education status of women educated outside the UK was equivalised to UK education levels, and as questions on women’s education status were slightly different in each cohort they were grouped to a comparable dichotomous measure of A-level or above and below A-level. Achieving A-level or above requires continuing in education post age 16 years, and this has been identified as a key measure of educational inequalities ( Tackey *et al.*, 2011).

### Analysis plan

We present a description of the characteristics of each cohort. We then look at consanguinity status by ethnicity for each cohort, illustrating any changes that occurred between the cohorts. Next we explore changes in consanguinity status for Pakistani heritage women. We estimate their rates of consanguinity by cohort characteristics with 95% confidence intervals: looking at age at pregnancy, self-reported financial status, and women’s education. We also look at rates by a combined measure of country of birth and age moved to the UK if women were born outside the UK. Results are presented in tables for each cohort and illustrated in figures comparing rates. This analysis enables us to determine how consanguinity status varies by these characteristics within each cohort, and also crucially what differences exist between the two cohorts; thereby allowing us to examine change in consanguinity status for Pakistani heritage women over time in a fixed geographical area.

After presenting these rates we then model consanguinity status. We employ multinomial regression models which look at consanguinity status for Pakistani heritage women, all data from both cohorts being combined in a single dataset for the analysis. First these models considered cohort, country of birth, women’s age at pregnancy, self-reported financial status, and women’s education individually in five separate univariable regression models. Then all five measures are included in a single multivariable regression model. In this section of the analysis, we used country of birth rather than the combined country of birth and age moved to UK measures, as age at pregnancy and age moved to UK from Pakistani are related (many women aged under 25 years at pregnancy could not have moved to the UK aged 25 and over). The univariable models are analogous with the descriptive analysis presented in Table 1. These univariable models are presented in order to compare them with the results of the multivariable model. Essentially the size of the effect for cohort in the univariable model estimates the observed differences between cohorts, and the size of the effect for cohort in the multivariable model estimates what the cohort effect would be if all other characteristics were held constant, i.e., controlling for the changes in these characteristics that occurred between the two cohorts. We also consider area level change that may have occurred between the time points of both cohorts. All statistical analysis was carried out using Stata 17 ( StataCorp, 2023).

**Table 1.** Cohort characteristics and consanguinity status for Pakistani heritage women. BiB, Born in Bradford; BiBBS, Born in Bradford’s Better Start.

	BiB in Better Start area					BiBBS				
Cohort characteristic		First cousins	Other blood relation	Not related			First cousins	Other blood relation	Not related	
	Total n	Percent	Percent	Percent	Total percent	Total n	Percent	Percent	Percent	Total percent
		(95% CI)	(95% CI)	(95% CI)			(95% CI)	(95% CI)	(95% CI)	
Age of woman at pregnancy (grouped)										
Under 25	508	42% (38%-46%)	23% (20%-27%)	35% (31%-39%)	100%	252	28% (23%-34%)	19% (15%-25%)	53% (47%-59%)	100%
25 to 29	546	41% (37%-45%)	22% (19%-25%)	37% (33%-41%)	100%	486	25% (21%-29%)	19% (15%-22%)	56% (52%-61%)	100%
30 to 34	363	37% (32%-42%)	22% (18%-27%)	41% (36%-46%)	100%	500	26% (23%-30%)	22% (19%-26%)	52% (47%-56%)	100%
35 and over	180	31% (24%-38%)	29% (23%-36%)	41% (34%-48%)	100%	313	30% (26%-36%)	16% (13%-21%)	53% (48%-59%)	100%
Combined country of birth and aged moved to UK										
Born UK	610	36% (32%-40%)	23% (20%-27%)	41% (38%-44%)	100%	707	20% (17%-23%)	16% (13%-19%)	64% (61%-68%)	100%
Born Pakistan: moved to UK under 16 yrs.	188	40% (33%-47%)	26% (20%-32%)	34% (27%-41%)	100%	131	33% (25%-41%)	17% (10%-23%)	50% (42%-59%)	100%
Born Pakistan: moved to UK 16 to 19 yrs.	226	51% (45%-58%)	21% (16%-27%)	27% (22%-33%)	100%	157	41% (34%-49%)	20% (14%-26%)	39% (31%-47%)	100%
Born Pakistan: moved to UK 20 to 24 yrs.	350	40% (35%-45%)	24% (19%-28%)	36% (31%-41%)	100%	300	36% (30%-41%)	25% (20%-30%)	39% (34%-44%)	100%
Born Pakistan: moved to UK 25 yrs. plus	171	30% (23%-37%)	22% (16%-28%)	49% (41%-56%)	100%	220	26% (20%-31%)	24% (18%-30%)	51% (44%-58%)	100%
Self-reported financial status										
Living comfortably	384	40% (35%-45%)	23% (19%-28%)	37% (32%-42%)	100%	608	27% (24%-31%)	22% (19%-26%)	51% (47%-55%)	100%
Doing alright	673	39% (35%-43%)	24% (21%-27%)	37% (34%-41%)	100%	593	27% (24%-31%)	19% (16%-22%)	54% (50%-58%)	100%
Just about getting by	398	39% (35%-44%)	25% (21%-30%)	36% (31%-41%)	100%	194	31% (25%-38%)	16% (11%-22%)	53% (46%-60%)	100%
Quite/ very difficult	127	38% (30%-47%)	14% (9%-21%)	48% (39%-57%)	100%	83	22% (14%-32%)	13% (7%-22%)	65% (54%-75%)	100%
Women’s education status										
A-level or higher	562	32% (28%-36%)	19% (15%-22%)	50% (46%-54%)	100%	714	23% (20%-26%)	17% (14%-20%)	60% (56%-64%)	100%
Lower than A-level	967	43% (40%-46%)	26% (23%-28%)	31% (28%-34%)	100%	769	30% (27%-33%)	21% (18%-24%)	49% (46%-52%)	100%

## Results

### Sample characteristics

Table 2 presents characteristics of the BiB cohort in the Better Start area and the BiBBS cohort. (For the sake of brevity, in the remainder of the text the ‘BiB cohort in the Better Start area’ will simply be referred to as the ‘BiB cohort’.) In both cohorts the majority of women were of Pakistani heritage, 64.8% and 61.6% in the BiB and BiBBS respectively. BiBBS women were older at the birth of their child, better educated and more financially secure.

**Table 2.** Characteristics of BiB cohort in the Better Start area, and BiBBS cohort. BiB, Born in Bradford; BiBBS, Born in Bradford’s Better Start.

Cohort characteristics	BiB in the Better Start area (n = 2,494)		BiBBS (n = 2,564)	
	n	Percentage (95% CI)	n	Percentage (95% CI)
Ethnicity				
White British	441	17.7% (16.3%-19.3%)	296	11.6% (10.4%-12.9%)
Pakistani heritage	1,615	64.8% (62.9%-66.7%)	1,571	61.6% (59.7%-63.5%)
Other ethnicity	435	17.5% (16.0%-19.0%)	683	26.8% (25.1%-28.5%)
Missing	3		14	
Age of woman at pregnancy				
Under 20	181	7.3% (6.3%-8.3%)	67	2.6% (2.1%-3.3%)
20 to 24	725	29.1% (27.3%-30.9%)	476	18.6% (17.1%-20.1%)
25 to 29	827	33.2% (31.3%-35.0%)	800	31.2% (29.4%-31.4%)
30 to 34	506	20.3% (18.8%-21.9%)	759	29.6% (27.9%-31.4%)
35 and over	255	10.2% (9.1%-11.5%)	462	18.0% (16.6%-19.5%)
Mean (SD)	26.9 (5.4)		29.3 (5.5)	
Whether born in UK				
No	1,288	51.7% (49.7%-53.6%)	1,382	54.1% (52.2%-56%)
Yes	1,205	48.3% (46.4%-50.3%)	1,173	45.9% (44%-47.8%)
Missing	1		9	
Self-reported financial status				
Living comfortably	579	23.5% (21.9%-25.2%)	877	35.9% (34.0%-37.9%)
Doing alright	1,013	41.1% (39.2%-43.1%)	995	40.8% (38.8%-42.7%)
Just about getting by	643	26.1% (24.4%-27.9%)	398	16.3% (14.9%-17.8%)
Quite difficult	174	7.1% (6.1%-8.1%)	127	5.2% (4.4%-6.2%)
Very difficult	56	2.3% (1.8%-2.9%)	44	1.8% (1.3%-2.4%)
Missing	29		123	
Women’s education status				
A-level or higher	850	36.5% (34.5%-38.4%)	1,061	44.8% (42.8%-46.8%)
Lower than A-level	1,481	63.5% (61.6%-65.5%)	1,307	55.2% (53.2%-57.2%)
Missing	163		196	

### Has there been a change in the number of consanguineous relationships recorded in the two cohorts?

**Figure 1.**
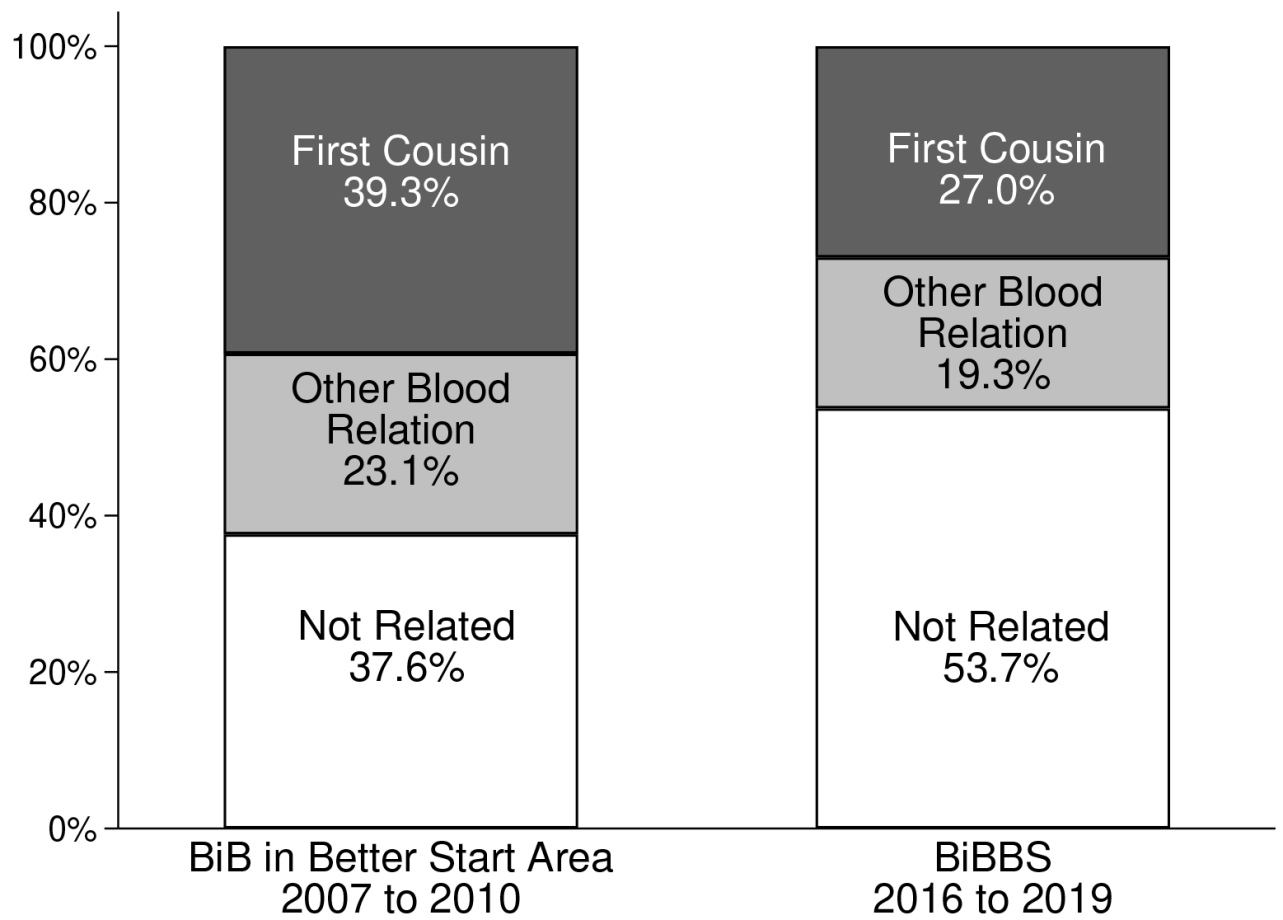
Consanguinity status for Pakistani heritage women in the BiB cohort in Better Start area and the BiBBS cohort. BiB, Born in Bradford; BiBBS, Born in Bradford’s Better Start.

Table 3 gives details of the rates of consanguinity for both cohorts by ethnicity. Rates in White British women were almost zero; and in “other ethnicity” women (not White British or Pakistani) they were similar in the two cohorts, 11.0% in BiB and 11.7% in BiBBS. Overall, there has been a substantial decrease in consanguineous relationships between the two survey periods consequent on reductions in the Pakistani heritage group. In the BiB cohort 37.6% of Pakistani heritage women were unrelated to the father of their child, and 53.7% were unrelated in the BiBBS cohort (see Figure 1)

**Table 3.** Consanguinity status for pregnancies by ethnicity in the BiB and BiBBS cohorts. BiB, Born in Bradford; BiBBS, Born in Bradford’s Better Start.

Ethnicity and consanguinity status	BiB		BiBBS	
	n	Percentage (95% CI)	n	Percentage (95% CI)
All				
Not related	1,429	57.8% (55.8%-59.7%)	1,724	68.4% (66.5%-70.1%)
First cousins	659	26.6% (24.9%-28.4%)	462	18.3% (16.9%-19.9%)
Other blood relation	386	15.6% (14.2%-17.1%)	336	13.3% (12.1%-14.7%)
Missing	20		42	
Total	2,494	100%	2,564	100%
White British				
Not related	439	99.8% (98.4%-100.0%)	294	99.7% (97.6%-100.0%)
First cousins	1	0.2% (0.0%-1.6%)	1	0.3% (0.0%-2.4%)
Other blood relation	0		0	
Missing	1		1	
Total	441	100%	296	100%
Pakistani heritage				
Not related	601	37.6% (35.5%-40.0%)	833	53.7% (51.2%-56.2%)
First cousins	627	39.3% (36.9%-41.7%)	418	27.0% (24.8%-29.2%)
Other blood relation	369	23.1% (21.1%-25.2%)	300	19.3% (17.4%-21.4%)
Missing	18		20	
Total	1,615	100%	1,571	100%
Other ethnicity				
Not related	387	89.0% (85.6%-91.6%)	593	88.4% (85.7%-90.6%)
First cousins	31	7.1% (5.1%-10.0%)	42	6.3% (4.7%-8.4%)
Other blood relation	17	3.9% (2.4%-6.2%)	36	5.4% (3.9%-7.4%)
Missing	0		12	
Total	435	100%	683	100%

### Consanguinity status of Pakistani heritage women by characteristics in both cohorts

We combined country of birth and the age that women who were born in Pakistan moved to the UK into a single measure, Table 4 indicates the numbers in these categories in each cohort. In the BiBBS cohort a higher proportion of Pakistani heritage women were born in the UK, and the women who were born in Pakistan were more likely to have moved to the UK at an older age.

**Table 4.** Combined country of birth and age moved to UK for Pakistani heritage women.

Combined country of birth and age moved to the UK for Pakistani heritage women	BiB		BiBBS	
	n	Percentage	n	Percentage
Born UK	610	39.5%	707	46.7%
Born in Pakistan: moved to UK aged under 16 yrs.	188	12.2%	131	8.6%
Born in Pakistan: moved to UK aged 16 to 19 yrs.	226	14.6%	157	10.4%
Born in Pakistan: moved to UK aged 20 to 24 yrs.	350	22.7%	300	19.8%
Born in Pakistan: moved to UK aged 25 yrs.	171	11.1%	220	14.5%
Born other country/ missing	52		36	
Total	1597	100.0%	1551	100.0%

**Figure 2.**
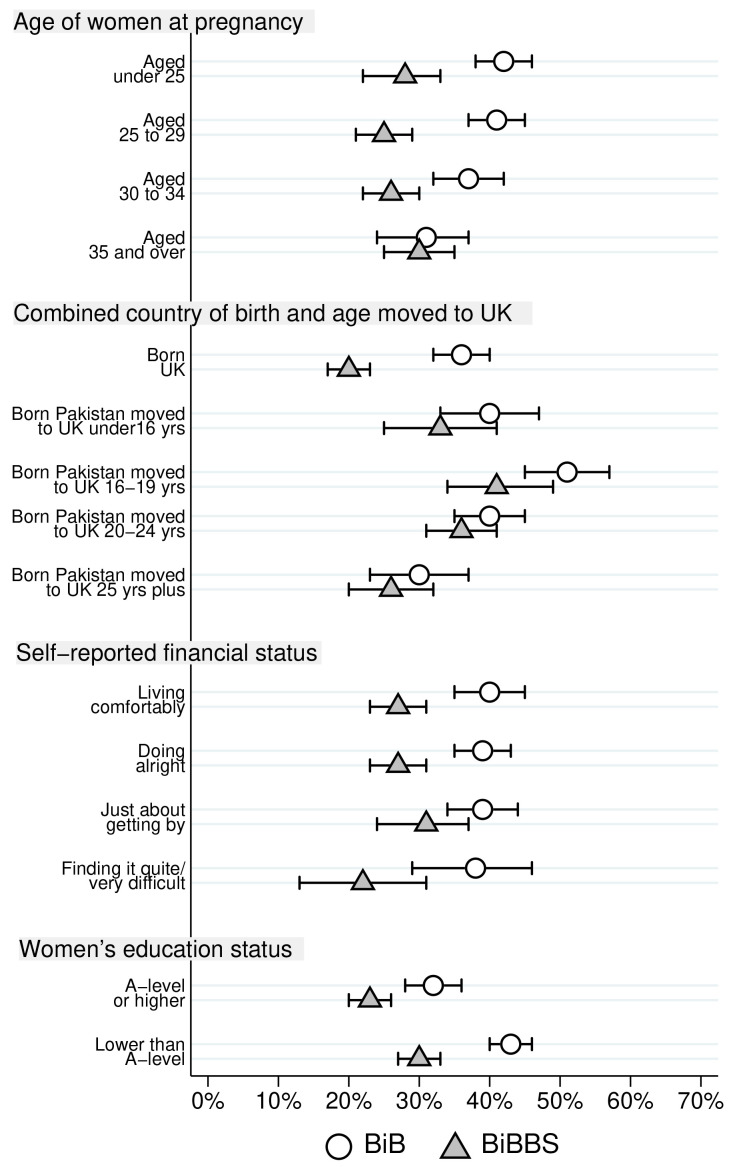
Percentage of Pakistani heritage women who are first cousins with the father of their child by cohort characteristics (with 95% confidence intervals).

**Figure 3.**
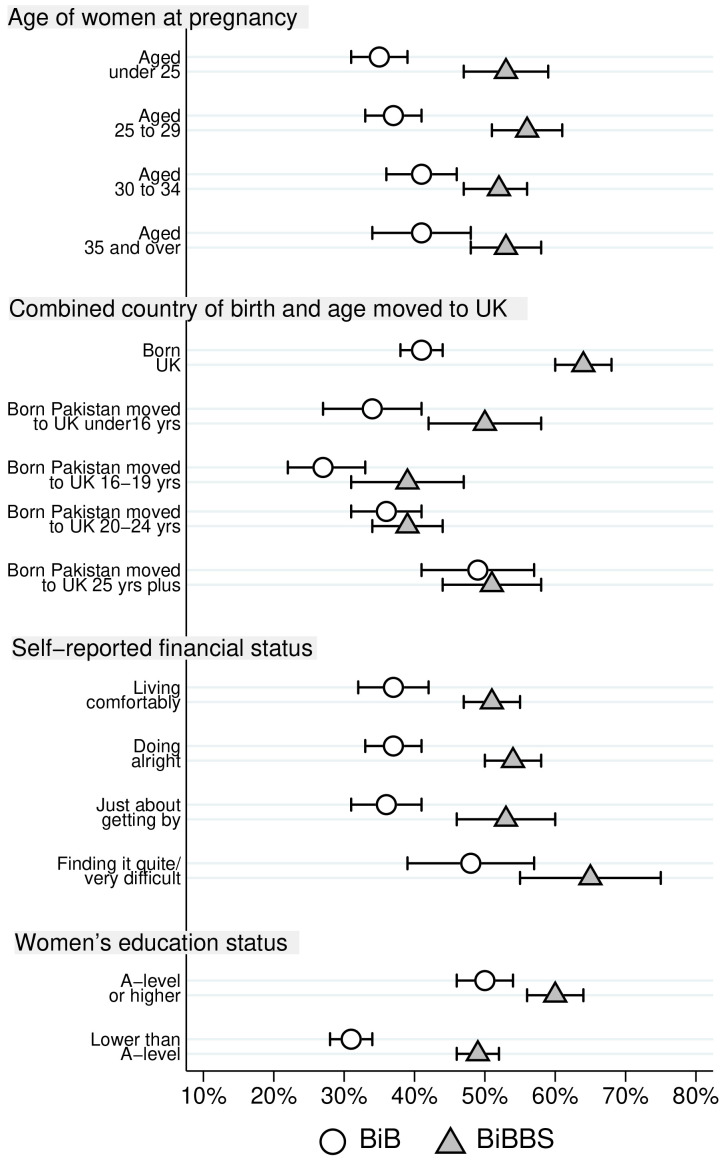
Percentage of Pakistani heritage women who are not related by blood to the father of their child by cohort characteristics (with 95% confidence intervals).

Differences in consanguinity status for Pakistani heritage women by characteristics are reported in Table 3 for both cohorts. The largest differences between the two cohorts are for first cousins and those not related by blood. For ease of comparison, proportions of Pakistani heritage women who were first cousins with the father of their child, and the proportion were not blood related are illustrated in Figure 2 and Figure 3 respectively.

Figure 2 illustrates differences in the proportion of first cousin relationships between the BiB and BiBBS cohort by characteristics, as well as illustrating the differences within each cohort. There was a lower proportion of first cousins in the BiBBS cohort for all age groups apart from women aged 35 years and over. The biggest difference was for the younger women. There is a substantial fall between the BiB and BiBBS cohort in the proportion of first cousin relationships in Pakistani heritage women who were born in the UK. There were also lower rates of first cousins for all women born in Pakistan regardless of the age they came to the UK, but these were small differences with overlapping 95% confidence intervals. The highest proportion of women who were in first cousin relationships was observed in women born in Pakistan who moved to the UK aged 16 to 19 years. There are differences between the cohorts for women who reported they were financially “living comfortably” and for those who reported they were 'doing alright' with the percentage in a first cousin union lower in BiBBS in both categories. Finally, Figure 2 illustrates that the proportion of Pakistani heritage women in first cousin relationships was lower for women with higher educational status within both cohorts. In terms of differences between cohorts, the BiBBS cohort had a lower proportion of women who were first cousins with the father of their child in both educational status groups.

Table 2 had indicated that overall, the proportion of Pakistani heritage women who were other blood relation with the father of their child was slightly lower in the BiBBS cohort, compared to the BiB cohort. Table 4 shows that there were no differences within the BiB cohort, apart from by women’s education, where fewer women with A-level or higher were other blood relations, compared to women who had lower than A-level education. There were no differences within the BiBBS cohort. There were only two differences between the BiB and BiBBS cohorts in the proportion of Pakistani heritage women who are other blood relation with the father of their child; fewer women aged 35 and over and fewer women born in the UK were other blood relations in BiBBS compared to BiB.

Figure 3 illustrates differences in the proportion of women not related by blood between the BiB and BiBBS cohort, and the differences within each cohort. There are relatively little differences by age group within each cohort, but large differences between the two cohorts. Figure 3 also illustrates that the largest difference between the two cohorts in the proportion not related by blood was for Pakistani heritage women born in the UK, and for women born in Pakistan who moved to the UK under the age of 16 years. There was no difference for women who were born in Pakistan who moved to the UK at an older age. The proportion of Pakistani heritage women not related by blood to the father of their child was higher in BiBBS than BiB for all self-reported financial status groups; these differences were substantive, apart from those finding it ‘quite/ very difficult’, where there were large overlapping confidence intervals for this group.

### Consanguinity status of Pakistani heritage women in the context of differences between cohorts

We saw in Table 2 that there were differences in the characteristics of women in the BiB and BiBBS cohort. (See Small *et al.*, 2024b for more detail, specifically the changes in age at pregnancy, country of birth, self-reported financial status, and education status broken down by ethnicity.) Compared to the BiB cohort Pakistani heritage women in the BiBBS cohort were more likely to be all of following; older at pregnancy, born in the UK, to self-report better financial status, and to have higher education status.

To what extent do these changes contribute to the observed differences in consanguinity status for Pakistani heritage women between the two cohorts? The results from multinomial regression models are presented in Table 5 as relative risk ratios (RRR) with 95 percent confidence intervals (95% CI). The relative risk ratios are presented in relation to the reference category for each variable; results for first cousin relationships and other blood relations are shown (the base reference outcome being women not related by blood to the father of their child).

**Table 5.** Results for separate univariable and combined multivariable multinomial regression models presented as relative risk ratios (base outcome = not related). BiB, Born in Bradford; BiBBS, Born in Bradford’s Better Start.

		Relative Risk Ratio (RRR)			
		Univariable		Multivariable	
		RRR	(95% CI)	RRR	(95% CI)
		First cousin			
Cohort					
	BiBBS (reference)	1.00		1.00	
	BiB in Better Start area	2.08	(1.77-2.45)	1.82	(1.51-2.18)
Combined country of birth and age moved UK					
	Born in UK (reference)	1.00		1.00	
	Not born in UK	1.84	(1.55-2.17)	1.68	(1.40-2.01)
Age of woman at pregnancy					
	Under 25 (reference)	1.00		1.00	
	25 to 29	0.80	(0.65-0.99)	0.88	(0.70-1.11)
	30 to 34	0.72	(0.57-0.89)	0.76	(0.59-0.98)
	35 and over	0.69	(0.53-0.89)	0.73	(0.54-0.98)
Self-reported financial status					
	Living comfortably (reference)	1.00		1.00	
	Doing alright	1.05	(0.86-1.27)	0.92	(0.75-1.13)
	Just about getting by	1.25	(0.99-1.58)	0.99	(0.77-1.28)
	Quite/ very difficult	0.82	(0.58-1.14)	0.61	(0.42-0.88)
Women’s education status					
	A-level or above (reference)	1.00		1.00	
	Lower than A-level	1.98	(1.68-2.35)	1.77	(1.48-2.12)
		Other blood relation			
Cohort					
	BiBBS (reference)	1.00		1.00	
	BiB in Better Start area	1.70	(1.42-2.05)	1.64	(1.33-2.02)
Whether born in UK					
	Born in UK (reference)	1.00		1.00	
	Not born in UK	1.58	(1.31-1.91)	1.49	(1.22-1.83)
Age of woman at pregnancy					
	Under 25 (reference)	1.00		1.00	
	25 to 29	0.82	(0.64-1.06)	0.93	(0.71-1.22)
	30 to 34	0.88	(0.68-1.13)	0.97	(0.74-1.29)
	35 and over	0.80	(0.60-1.08)	0.92	(0.66-1.28)
Self-reported financial status					
	Living comfortably (reference)	1.00		1.00	
	Doing alright	0.95	(0.76-1.18)	0.86	(0.69-1.08)
	Just about getting by	1.08	(0.83-1.40)	0.85	(0.63-1.13)
	Quite/ very difficult	0.51	(0.33-0.79)	0.40	(0.25-0.63)
Women's education status					
	A-level or above (reference)	1.00		1.00	
	Lower than A-level	1.88	(1.55-2.28)	1.76	(1.44-2.16)

In the separate univariable model Pakistani heritage women in the BiB cohort had twice the probability of being first cousins with the father of their child compared to the BiBBS cohort. This ratio reduced very slightly in the multivariable models, after controlling for other characteristics. Also, Pakistani heritage women in the BiB cohort had a higher probability of being other blood relations in the univariable models compared to the BiBBS cohort; this also remained similar in the multivariable models after controlling for other measures.

For the other measures the model results are similar to the results observed in the analysis previously presented, except for a finding related to self-reported financial status. In the multivariable models those who reported that their financial status was quite or very difficult had a lower probability of being first cousin and other blood relation compared to those who reported ‘living comfortably’. Previous descriptive analysis had suggested women who reported finding their financial situation ‘quite/ very difficult’ were less likely to be related by blood to the father of their child (see Figure 3), but these differences were not conclusive due to large confidence intervals. The analysis utilising regression models combine both cohorts into a single dataset, and so have more power to detect these differences.

To summarise, when we looked at multinominal regression models examining the probability of Pakistani heritage respondents being in consanguineous relationships, considering differences by cohort and by what age respondents came to the UK, we found that consanguineous relationships were highest amongst those born outside the UK who moved to the UK when aged between 16 and 19 years of age: the lowest rates were for those born in the UK.

We found that between the BiB and BiBBS cohorts, there was a large increase in the percentage of Pakistani heritage respondents born in the UK who were not related (from 40.5% to 64.2%), and a smaller increase for Pakistani heritage respondents born outside the UK but who came to the UK before the age of 16 (from 33.5% to 50.4%). There were also increases for Pakistani heritage respondents who were born outside the UK and came to the UK after the age of 16 years, but these were small, with overlapping confidence intervals. For first cousin relationships there was an overall reduction between the two cohorts, driven by a large reduction amongst Pakistani heritage respondents born in the UK (from 36.2% to 19.8%).

### Area level change

Around 50% of the White British children born in the Better Start area had moved out by the age of 5, compared to 15% of Pakistani heritage children. These high levels of outward migration should be seen in the context of the Better Start area being relatively small geographically, with a population of about 60,000 people (see Small *et al.*, 2024b). White British families are poorer and are more transient than the Pakistani heritage families. This relative stability suggests that the Pakistani heritage community in the Better Start area may be more comparable over time than the White British group, and certainly more comparable than the diverse “other ethnicity” group.

## Discussion

### Efforts to raise awareness of genetic risk

Throughout the period covered by BiB and BIBBs health commissioners and providers in Bradford have been well aware of the high rates of recessive disorders and their attendant impacts on mortality and morbidity and they had been concerned to develop health promotion, heath education and care initiatives in response (see reports from The City of Bradford Joint Strategic Needs Assessments – www.jsna.bradford.gov.uk and from the City of Bradford Child Death Overview Panel – www.bradford.moderngov.co.uk). However, the Department of Health ( 2010 and 2012) and Khan *et al.* (2010) report that knowledge of genetic risk and service uptake among communities at higher risk of recessive conditions due to consanguinity was poor. By 2016 there was more attention being paid to local initiatives where responses to consanguinity and genetic risk had been developed ( Salway *et al.*, 2016) and moves to build a consensus about what health policy and practice was required were underway at a national level ( Salway *et al.*, 2019).

In Bradford there was considerable publicity across a range of media, including local media, attendant on the publication of Born in Bradford’s paper about rates of recessive disorder and links to consanguinity in the BiB cohort ( Sheridan *et al.*, 2013: https://www.bbc.co.uk/news/uk-england-leeds-23183102.) The size and longevity of BiB means it is well-known in the city - in the years BiB recruited to the study over half the age cohort of children in the city were participants, as were their mothers and some of their fathers. As well as contact to collect data there were regular updates on research findings sent to participant families and many meetings and events where the messages from BiB were shared with the wider metropolitan population. A research study with reach such as this will impact on levels of knowledge about risks to health and may change choices people make about health related behaviours (see Quick *et al.*, 2017). As well as a possible direct effect, BiB also encourages and supports local health services to address issues it has highlighted and provides robust local data to underpin their work. Voluntary sector and faith based organisations also become better informed about the issues. BiB may then be both a direct link to change and a catalyst for change in other organisations policies and priorities and this, in turn, contributes to extending informed choice in the community, enhancing healthy choices and supporting evidence based health provision.

Our data is drawn from a specific area of one city in the north of England, and that city has been the site of this enhanced engagement with understandings of genetic risk. In consequence the choices made by its Pakistani heritage community may be specific to residents of this city and not generalisable to other communities of Pakistani heritage in the UK and elsewhere.

### Some potential weaknesses in our data can be noted

Consanguinity is self-reported. There was a high level of completion of questions about consanguinity, indicating that respondents knew their status and were willing to share it. Interviewers were trained and experienced and, in BiB, some family trees were also collected from study recruits and, when analysed, these showed a close link with questionnaire data ( Sheridan *et al.*, 2013). It is unlikely that any over or under reporting of consanguinity status would vary between the two time periods our cohorts draw on. Biraderi was not asked in the BiBBS survey so is not considered in the models considering differences between the cohorts (see Bittles & Small, 2016: and Small *et al.* (2017) on the importance of biraderi as an example of endogamy). Age at marriage, year of marriage or age/year when they entered a relationship with their partner was not asked in BiB or BiBBS. We also do not know year of betrothal – i.e., the year when decisions were made to marry a cousin or to marry a non-related person. We have information about previous parity, but not about the date of birth of the respondents first child (if they have children prior to the pregnancy that was included in each study).

### Two cohort analysis

An innovative aspect of our approach is the use of two cohorts recruited from the same geographical area but separated by time. For this approach to be of maximum value we must identify that the areas and the characteristics of the population of interest stayed similar and there was continuity in the broad details of the wider social, political and economic context. Small *et al.*, 2024b provides some Census data from 2011 and 2022 in order to situate our findings within this wider context. We would argue that our approach expands the methodological possibilities for longitudinal research. Wadsworth and Bynner (2011) discuss how cohort studies in different time periods can give an indication about patterns of social change. Design of longitudinal studies which compare variables of interest across separate cohorts is greatly aided by using standardised data collection approaches, as in the cohorts we draw on. Comparing cohorts also extends the uses of valuable data willingly given by cohort members.

### Reasons for reductions in consanguinity

Our data suggests some key areas to be explored but, in itself, does not explain why there has been a reduction. We have carried out qualitative research to explore reasons why members of the Pakistani heritage community in the city think rates of consanguinity might have changed over time. This qualitative research considers involvement in education, changes in migration patterns and differences in partner choices depending on age of women alongside other factors that were raised by our participants. We also looked at more distal factors including changes in economic wellbeing and shifts in the law and regulations shaping migration to the UK ( Small *et al.*, 2024c).

Data collection for the BiBBS cohort finished in November 2019. The distribution of changes in rates of consanguinity by age of women, with larger reduction in the rates of consanguineous relationships for women giving birth at a younger age and relatively stable rates in women giving birth at an older age, may indicate that the falls we report are part of a social change in Bradford’s Pakistani heritage population and a concomitant downward trajectory in consanguinity. If this is the case by the date of publication we might expect lower rates than we report from births up to 2019.

## Conclusions

In a relatively short time period, there has been a drop in the proportion of Bradford women of Pakistani heritage in consanguineous relationships. We have found this out by looking at two groups from the same small geographical area, each recruited into ongoing cohort studies. The area they are recruited from has changed in some of its characteristics – but the change for women of Pakistani heritage has been of a degree that allows us to be confident we are comparing like-with-like groups.

A majority of Pakistani heritage women in BiB were in consanguineous relationships (62.4%). In BiBBS Pakistani heritage women in consanguineous relationships were in a minority (46.3%). This is a substantial drop in a long-established practice in just nine years. Drops in consanguinity are linked with changes in the rates of consanguinity in women born in the UK or who came to the UK as children. A change in numbers educated to A level and beyond is important. Drops are large in Pakistani heritage women under 25 years at the birth of their child where the fall in first cousin unions is from 41.9% to 27.8%. Consanguinity rates in the over 35’s were about the same in both cohorts.

Knowledge of consanguinity prevalence and trends, and the detailed characteristics of who is in consanguineous unions, helps identify at-risk populations, and this can be used to enhance risk awareness and to help target genetic counselling.

The identification of the reductions in consanguinity we report, in particular the considerable reductions in younger people, underscore a complex interplay of cultural, social, and demographic factors. It may be that we are seeing generational changes, and newly evolving societal norms. But these changes need to be monitored to see if they are indications of a lasting change and they need to be considered in other settings where consanguinity is common to see how widespread these reductions in consanguinity are. 

A drop in the prevalence of consanguinity is likely to lead to a reduction in recessive genetic disorders and in the high rates of morbidity and mortality evident in children of consanguineous parents in Bradford. If the drop we have identified is replicated in other areas with Pakistani heritage communities our findings will be significance for families from these communities and also for health planning and care provision.

## Ethics and consent

Ethical approval has been obtained from the Bradford Research Ethics Committee (Ref 07/H1302/112) for BiB (approval letter dates 1/4/2008). and by Bradford Leeds NHS Research Ethics Committee (15/YH/0455) for BiBBS (approval letter dated 2/11/2015). Research governance approval has been provided from Bradford Teaching Hospitals NHS Foundation Trust. All participants in both cohorts were given Participant Information Sheets approved by the respective ethics committees before recruitment and all participants signed Consent Forms that included consent for data storage, data usage and data sharing.

## Data Availability

Researchers are encouraged to make use of the BiB and BiBBS data, which are available through a system of managed open access. Before you contact us, please make sure you have read our Guidance for Collaborators. Our BiB Executive reviews proposals on a monthly basis and we will endeavour to respond to your request as soon as possible. You can find out about the different datasets in our Data Dictionary. If you are unsure if we have the data that you need, please contact a member of the BiB team (borninbradford@bthft.nhs.uk). Once you have formulated your request please complete the ‘Expression of Interest’ form available here and send to borninbradford@bthft.nhs.uk. If your request is approved we will ask you to sign a Data Sharing Contract and a Data Sharing Agreement, and if your request involves biological samples we will ask you to complete a material transfer agreement. Harvard Dataverse: Changes in prevalence and patterns of consanguinity in Bradford, UK – evidence from two cohort studies: Extended material. https://doi.org/10.7910/DVN/F57B2I ( Small *et al.*, 2024b). Extended data contains additional analysis, providing contextual data of the study population, including 2011 and 2021 UK Census data comparing the populations in the study area to the wider Bradford area and England as a whole. We ensured our reporting met the STROBE guidelines for observational studies. Specifically, we have used the cross sectional STROBE guidelines, rather than the cohort guidelines, as our paper essentially uses a comparison between two cross sectional datasets ( von Elm *et al.*, 2007). Data are available under the terms of the Creative Commons Zero "No rights reserved" data waiver (CC0 1.0 Public domain dedication).

## References

[ref-1] Bishop CF Small N Parslow R : Healthcare use for children with complex needs: using routine health data linked to a multiethnic, ongoing birth cohort. *BMJ Open.* 2018;8(3): e018419. 10.1136/bmjopen-2017-018419 29525769 PMC5855244

[ref-2] Bittles AH : Consanguinity in context.Cambridge, Cambridge University Press,2012. 10.1017/CBO9781139015844

[ref-3] Bittles AH Small NA : Consanguinity, genetics and definitions of kinship in the UK Pakistani population. *J Biosoc Sci.* 2016;48(6):844–854. 10.1017/S0021932015000449 26707179

[ref-4] Clark DW Okada Y Moore KHS : Associations of autozygosity with a broad range of human phenotypes. *Nat Commun.* 2019;10(1): 4957. 10.1038/s41467-019-12283-6 31673082 PMC6823371

[ref-5] Department of Health: Tackling health inequalities in infant and maternal health outcomes: report of the Infant Mortality National Support Team.Health Inequalities Unit. London. Department of Health,2010. Reference Source

[ref-6] Department of Health: Building on our inheritance: genomic technologies in healthcare.Human Genomics Strategy Group. London, Department of Health,2012. Reference Source

[ref-7] Dickerson J Bird P McEachan R : Born in Bradford’s Better Start: an experimental birth cohort study to evaluate the impact of early life interventions. *BMC Public Health.* 2016;15(1): 711. 10.1186/s12889-016-3318-0 27488369 PMC4996273

[ref-8] Dickerson J Bridges S Willan K : Born in Bradford's Better Start (BiBBS) interventional birth cohort study: interim cohort profile [version 1; peer review: 1 approved, 1 approved with reservations]. *Wellcome Open Res.* 2022;7:224. 10.12688/wellcomeopenres.18394.1 37830108 PMC10565418

[ref-10] Khan N Benson J MacLeod R : Developing and evaluating a culturally appropriate genetic service for consanguineous South Asian families. *J Community Genet.* 2010;1(2):73–81. 10.1007/s12687-010-0012-2 22460207 PMC3185987

[ref-11] Lodh R Hou B Hough A : Health care utilisation and education outcomes of children with rare diseases: a born in Bradford cohort study. *Eur J Pediatr.* 2023;182(12):5511–5517. 10.1007/s00431-023-05225-4 37782349

[ref-12] Malawsky DS van Walree E Jacobs BM : Influence of autozygosity on common disease risk across the phenotypic spectrum. *Cell.* 2023;186(21):4514–4527. e14. 10.1016/j.cell.2023.08.028 37757828 PMC10580289

[ref-13] Quick A Böhnke JR Wright J : Does involvement in a cohort study improve health and affect health inequalities? A natural experiment. *BMC Health Serv Res.* 2017;17(1): 79. 10.1186/s12913-017-2016-7 28122612 PMC5264453

[ref-14] Raynor P , The Born in Bradford Collaboratie Group : Born in Bradford, a cohort study of babies born in Bradford, and their parents: protocol for the recruitment phase. *BMC Public Health.* 2008;8: 327. 10.1186/1471-2458-8-327 18811926 PMC2562385

[ref-15] Salway S Ali P Ratcliffe G : Responding to the increased genetic risk associated with customary consanguineous marriage among minority ethnic populations: lessons from local innovations in England. *J Community Genet.* 2016;7(3):215–228. 10.1007/s12687-016-0269-1 27311843 PMC4960028

[ref-16] Salway S Yazici E Khan N : How should health policy and practice respond to the increased genetic risk associated with close relative marriage? Results of a UK Delphi consensus building exercise. *BMJ Open.* 2019;9(7): e028928. 10.1136/bmjopen-2019-028928 31289086 PMC6615806

[ref-17] Sheridan E Wright J Small N : Risk factors for congenital anomaly in a multiethnic birth cohort: an analysis of the Born in Bradford study. *Lancet.* 2013;382(9901):1350–9. 10.1016/S0140-6736(13)61132-0 23830354

[ref-19] Small N Bittles ES Petherick E : Endogamy, consanguinity and the health implications of changing marital choices in the UK Pakistani community. *J Biosoc Sci.* 2017;49(4):435–446. 10.1017/S0021932016000419 27573732

[ref-25] Small N Kelly B Malawsky DS : Mortality, morbidity and educational outcomes in children of consanguineous parents in the Born in Bradford cohort [version 2; peer review: 2 approved, 1 approved with reservations]. *Wellcome Open Res.* 2024a;9:319. 10.12688/wellcomeopenres.22547.2 39372840 PMC11452767

[ref-18] Small N Kelly B Wright J : Changes in prevalence and patterns of consanguinity in Bradford, UK - evidence from two cohort studies: Extended material. *Harvard Dataverse.* [Dataset].2024b. 10.7910/DVN/F57B2I PMC11809158 39931108

[ref-27] Small N Razaq R Sharma V : Changing patterns in marriage choice and related health risk in the Pakistani heritage community in Bradford UK: a qualitative study [version 1; peer review: awaiting peer review]. *Wellcome Open Res.* 2024c;9:690. 10.12688/wellcomeopenres.23338.1 PMC12120418 40444146

[ref-20] StataCorp: Stata Statistical Software: Release 17.College Station, TX: StataCorp LLC,2023.

[ref-21] Tackey ND Barnes H Khambhaita P : Poverty, ethnicity and education.York: Joseph Rowntree Foundation,2011. Reference Source

[ref-9] von Elm E Altman DG Egger M : The Strengthening the Reporting of Observational Studies in Epidemiology (STROBE) statement: guidelines for reporting observational studies. *Lancet.* 2007;370(9596):1453–7. 10.1016/S0140-6736(07)61602-X 18064739

[ref-22] Wadsworth M Bynner J : A companion to life course studies.London, Routledge,2011. Reference Source

[ref-23] Wright J Small N Raynor P : Cohort profile: the Born in Bradford multi-ethnic family cohort study. *Int J Epidemiol.* 2013;42(4):978–991. 10.1093/ije/dys112 23064411

