# Supplementary material for: Changes in prevalence and patterns of consanguinity in Bradford, UK  – evidence from two cohort studies
Source: Wellcome Open Res. 2024 Nov 28;9:222. Originally published 2024 Apr 24. [Version 2] doi: 10.12688/wellcomeopenres.21121.2 (PMC11809158; doi:10.12688/wellcomeopenres.21121.2)
Supplement: Peer review history

# Reviewer report: Reviewer response for version 2

**DOI:** 10.21956/wellcomeopenres.25901.r115504

**Published:** 2025-01-27

**Authors:** Babak Khoshnood

**Affiliations:** 1 INSERM Centre de Recherche Épidémiologie et Statistique, Paris, France

Thank you for the opportunity to read and review this interesting manuscript. The article has already gone through a couple of reviews and I find the current version an accurate and well-written report of an interesting and useful study. I only have a few remarks, which I hope the authors find helpful in possibly revising the article.

 There is mention that the overall risk of congenital anomalies (CA) was twice as high for newborns of mothers in consanguineous couples than those who were not (6 vs. 3 %). This requires some clarification and explanation as follows: 

 Are these total or live birth prevalence figures? If they are total prevalence figures the finding is at least somewhat surprising. As the authors point out, the risk of (autosomal) recessive genetic disorders is higher (twice as high) in cases of consanguinity. However, to my knowledge, this is not true of other anomalies including those without clearly known genetic inheritance patterns. Hence, one would not expect a two-fold increase in the OVERALL risk of CA associated with consanguinity.

 If these figures refer instead to live birth prevalence, one possible / partial explanation for the two-fold difference may be that in addition to consanguinity resulting in a two-fold higher prevalence of recessive genetic disorders, due to associations perhaps with socioeconomic factors including maternal education, the probability of prenatal diagnosis and Terminations of Pregnancy for Fetal Anomalies (TOPFA) may be different in relation to consanguinity. Therefore, the two-fold higher OVERALL risk of CA may be due to higher TOPFA for cases not associated with consanguinity.

 A perhaps useful "back of the envelope" calculation of expected changes in attributable fractions in the risk of CA related to consanguinity as a result of the decrease in consanguineous unions may make a useful addition to the paper but I defer to authors as to whether this is really necessary.

Is the work clearly and accurately presented and does it cite the current literature?

Yes

If applicable, is the statistical analysis and its interpretation appropriate?

Yes

Are all the source data underlying the results available to ensure full reproducibility?

Yes

Is the study design appropriate and is the work technically sound?

Yes

Are the conclusions drawn adequately supported by the results?

Yes

Are sufficient details of methods and analysis provided to allow replication by others?

Yes

Reviewer Expertise:

Epidemiology of congenital anomalies, in particular Down syndrome, neural tube and congenital heart defects

I confirm that I have read this submission and believe that I have an appropriate level of expertise to confirm that it is of an acceptable scientific standard.

# Reviewer report: Reviewer response for version 2

**DOI:** 10.21956/wellcomeopenres.25901.r115509

**Published:** 2025-01-02

**Authors:** Grace (Qun) Miao

**Affiliations:** 1 Better Outcomes Registry & Network (BORN) Ontario, Children’s Hospital of Eastern Ontario, Ottawa, Canada

It is a interesting topic. The study was well designed and well written. The authors have made revisions to the manuscript. The quality has been improved significantly. I don't have any comments. It is ready for indexing. The table and graphs presented clearly. The conclusions drawn adequately supported by the results.

Is the work clearly and accurately presented and does it cite the current literature?

Yes

If applicable, is the statistical analysis and its interpretation appropriate?

Yes

Are all the source data underlying the results available to ensure full reproducibility?

Yes

Is the study design appropriate and is the work technically sound?

Yes

Are the conclusions drawn adequately supported by the results?

Yes

Are sufficient details of methods and analysis provided to allow replication by others?

Yes

Reviewer Expertise:

maternal and child health

I confirm that I have read this submission and believe that I have an appropriate level of expertise to confirm that it is of an acceptable scientific standard.

# Reviewer report: Reviewer response for version 2

**DOI:** 10.21956/wellcomeopenres.25901.r115510

**Published:** 2025-01-02

**Authors:** Julian Little

**Affiliations:** 1 Epidemiology & Community Medicine, University of Ottawa, Ottawa, Canada

This manuscript reports on the prevalence of consanguinity in two sets of participants from the Bradford area, one recruited in 2007-2010, the other in 2016-9. A decline in prevalence was reported. The earlier data set was larger because it covered a larger geographical area. The analysis in the present manuscript was restricted to cover the same geographical sub area in which the second set of participants were recruited. Reference is given to another paper in which the subset and total population in the first period are compared - it would have been helpful to present summary of the results of the comparison.

 The manuscript seems timely as consanguinity has been discussed in the UK Parliament recently (articles by Matthew Syed, The Times, 15 December 2024 and 9 April 2024) and there have been moves to pass legislation in some Scandinavian countries ( https://www.dailymail.co.uk/news/article-13954609/Sweden-Denmark-ban-marriage-cousins.html)

 In the 2nd paragraph of the data subsection of the methods, It is stated that women who did not give full answers to questions about consanguinity were excluded from analysis. How many were excluded in each set of data? What was the pattern of missingness?

 In the introduction, there is a statement that “there were other significant factors associated with” congenital anomalies - what were these, and what was the magnitude of association?

 My understanding is that immigrants from Pakistan tended to come from the same geographical area, and then settle in the same area in the UK. This would likely have limited mating choices in a group that strongly encourages family creation in the same culture. Is there any evidence that this has changed between the two periods. Did you collect any information on area of birth? If so, might this be more diverse in the more recent group? Within the study area, do you know if one or both partners had moved from elsewhere in Bradford (or the UK) into the area, and if this differed between the two periods?

Is the work clearly and accurately presented and does it cite the current literature?

Partly

If applicable, is the statistical analysis and its interpretation appropriate?

Partly

Are all the source data underlying the results available to ensure full reproducibility?

Partly

Is the study design appropriate and is the work technically sound?

Partly

Are the conclusions drawn adequately supported by the results?

Partly

Are sufficient details of methods and analysis provided to allow replication by others?

Partly

Reviewer Expertise:

Epidemiology

I confirm that I have read this submission and believe that I have an appropriate level of expertise to confirm that it is of an acceptable scientific standard, however I have significant reservations, as outlined above.

## References

[1] Sweden and Denmark both move to ban marriage between cousins over fears the practice breeds domestic violence. Reference source

# Reviewer report: Reviewer response for version 2

**DOI:** 10.21956/wellcomeopenres.25901.r113710

**Published:** 2024-12-13

**Authors:** Parveen Ali

**Affiliations:** 1 University of Sheffield, Sheffield, UK

Thank you very much for considering and incorporating review.

Is the work clearly and accurately presented and does it cite the current literature?

Partly

If applicable, is the statistical analysis and its interpretation appropriate?

Yes

Are all the source data underlying the results available to ensure full reproducibility?

No source data required

Is the study design appropriate and is the work technically sound?

Yes

Are the conclusions drawn adequately supported by the results?

Yes

Are sufficient details of methods and analysis provided to allow replication by others?

Yes

Reviewer Expertise:

Inequalities in Health related to gender and ethnicity

I confirm that I have read this submission and believe that I have an appropriate level of expertise to confirm that it is of an acceptable scientific standard.

# Reviewer report: Reviewer response for version 2

**DOI:** 10.21956/wellcomeopenres.25901.r113890

**Published:** 2024-12-12

**Authors:** Atta Ur Rehman

**Affiliations:** 1 Hazara University, Mansehra, Khyber Pakhtunkhwa, Pakistan

The authors have compared the prevalence of consanguinity in two independent cohorts (2007-2010; 2016-2019) from Bradford, England separated by a nine-years gap. Their findings suggest a substantial decline in consanguinity among women of Pakistani heritage over the years. For instance, first-cousin marriages dropped from 39.3% to 27.0%, and other blood relation marriages decreased from 23.1% to 19.3%. Alternatively, the rate of unrelated marriages increased from 37.6% to 53.7% among Pakistani heritage women.

 Findings of this study underscore the importance of recognizing shifts in consanguinity rates and associated health needs for planning antenatal, pediatric, and genetic services in Bradford and similar communities. The observed trends may reflect broader changes in partner selection within these populations. In general, the authors have prepared a well-structured manuscript with clear presentation of objectives and results. However, I have a few comments:

**Introduction: **The authors have reasonably provided basic knowledge about consanguinity, its global prevalence, and correlation of consanguinity with congenital anomalies especially recessive disorders. However, I would suggest the authors to briefly discuss about the recent patterns/trends in consanguinity in native Pakistani population. This will be integral for findings of this study because a recent study  [PMID: 38314634] has shown a temporal decline in consanguinity in Pakistan which is quite similar to findings of this study.

**Methods:** Since consanguinity rates are not uniform across Pakistan and are influenced by many variables, it would be more interesting if the authors could provide data or at least mention about the various sociodemographic attributes of their respondents including ethnicities, language, geographic location etc.

**Results:** The results are self-explanatory; thus, I suggest no changes.

**Discussion:** The authors need a more comprehensive discussion on their findings in the context of Pakistani population since the respondents are largely of Pakistani heritage. Also, the authors need to fully appreciate regional differences in consanguinity rates within Pakistani community, especially by identifying regions where highest rates of consanguinity has been reported. The authors need to associate their findings with the recent trends in consanguinity in Pakistan (For details, please refer to:  PMID: 38314634).

Is the work clearly and accurately presented and does it cite the current literature?

Partly

If applicable, is the statistical analysis and its interpretation appropriate?

Yes

Are all the source data underlying the results available to ensure full reproducibility?

Yes

Is the study design appropriate and is the work technically sound?

Yes

Are the conclusions drawn adequately supported by the results?

Yes

Are sufficient details of methods and analysis provided to allow replication by others?

Yes

Reviewer Expertise:

My interest is at the interface between human genetics and epidemiology.

I confirm that I have read this submission and believe that I have an appropriate level of expertise to confirm that it is of an acceptable scientific standard, however I have significant reservations, as outlined above.

## References

[1] : Consanguinity in northwest Pakistan: evidence of temporal decline. 10.1017/S0021932024000026 10.1017/S0021932024000026 38314634

# Reviewer report: Reviewer response for version 1

**DOI:** 10.21956/wellcomeopenres.23363.r107687

**Published:** 2024-11-07

**Authors:** Anisa Bibi

**Affiliations:** 1 Quaid-i-Azam University, Islamabad, Pakistan

**Summary:**

 This article uses information from two important cohort studies to examine the prevalence and changing trends of consanguineous marriages in Bradford, UK. Marriage between relatives, or consanguinity, is a social phenomena that affects genetic health, especially in communities where consanguinity is more common. By contrasting data from two cohorts gathered at various points in time, the authors hope to provide a thorough examination of consanguinity patterns in Bradford and gauge shifts in prevalence and attitudes.

 Given Bradford's distinct demographic profile—which includes a sizable population of South Asian heritage, where consanguinity has long been practiced—the question is quite pertinent. In a healthcare setting where genetic risks related to consanguinity are of concern, the study offers important insights into how cultural practices change over time within immigrant populations.

**Title and abstract: **The title is appropriate and sums up the primary focus of the research. The abstract clearly conveys the main findings, methodology, and goals of the study and is well-structured. Clarity would be improved by including a brief overview of the study's limitations.

**Introduction: **The significance of researching consanguinity in Bradford is clearly stated in the introduction, which also highlights the ramifications for culture and health. It highlights a knowledge gap about the temporal variations in consanguinity prevalence and patterns and gives a good summary of earlier research. The stated goals of the study are well-aligned with the background information. The case for this study might be strengthened, nevertheless, by additional discussion of the possible genetic consequences and public health significance.

**Methods:**

 Cohort studies are suitable for longitudinal studies.  

 The background information is sufficient, and the cohort, inclusion, and exclusion criteria employed provide accurate representations of the Bradford community. The data collection procedure was suitable. The statistical techniques were appropriate and sufficient. The pattern and data analysis were satisfactory.  They talk about the limitations and possible biases in consanguinity data.

**Results:**

 Every cohort study is covered sequentially, and the results are arranged rationally.  Key data has been presented by the writers using tables and figures in an efficient manner, and the text well supports them.The authors provide adequate context for readers to grasp the findings' importance, especially when comparing data from two different cohort studies. The results directly address the main objective of the paper—understanding changes in the prevalence and patterns of consanguinity over time.The statistical methods employed to examine the data are suitable for the kind of data generated and the study design. When applicable, the authors report statistical significance and include metrics such as confidence intervals to show how accurate their predictions are. The data was well interpreted. The conclusions about changes in consanguinity patterns justified by the data and author acknowledge the potential biases. 

 Bradford has a distinct demographic with a significant South Asian community, making it a good place to examine consanguinity patterns because consanguineous marriages are more prevalent there. Understanding the genetic and societal factors that may affect public health and the need for genetic counseling will be improved by the current study.

**Discussion and conclusion:**

 There is a clear fall in first cousin marriage**** from 39.3% to 27.0% and  those who were of other blood relations fell from 23.1% to 19.3%. The study highlighted ethnic disparities, showing that consanguinity remains more common among South Asian communities compared to the general population. However, there was also an observed increase in consanguineous marriages in other ethnic groups over time. 

 Younger individuals and those with higher educational attainment were less likely to engage in consanguineous marriages, while consanguinity was more common among older generations and those with lower educational levels.

 The study stresses the potential public health implications of high consanguinity rates, such as the increased risk of autosomal recessive genetic disorders. It calls for improved genetic counseling and screening services for affected populations. The research indicates a shift in marriage patterns in the South Asian community, with a higher rate of consanguinity in later generations, possibly influenced by changing social norms and migration patterns.

 These findings underscore the complex interplay of cultural, social, and demographic factors influencing consanguinity and its potential health impacts in the context of a multicultural society like Bradford.

**Key strengths:**

**Longitudinal Approach: **By comparing two datasets from several eras, the study provides an insightful look at consanguinity trends across time. This method enables trend analysis, which may shed light on the consequences of acculturation, generational changes, and evolving societal norms.

**Population Significance: **Bradford is a good place to study consanguinity because of its large proportion of people of South Asian heritage. The results could influence community participation tactics, genetic counseling programs, and regional health policy.

**Methodological Rigor: **The study makes use of strong statistical analyses, such as comparisons of the prevalence of consanguinity and its relationship to sociodemographic variables. The study is more reliable and reproducible because the authors have provided a comprehensive explanation of their methods.

**Relevance to Clinical and Public Health:** Knowledge of consanguinity prevalence and trends helps identify at-risk populations, and these results can be used directly to enhance risk awareness and genetic counseling in healthcare.

**Areas for improvement:**

**Clarification of Cohort Differences:** Despite the fact that two cohorts were examined, a more thorough comparison of the cohorts' sample attributes, recruiting strategies, and potential confounders will enhance the article. The comparability of the datasets and any potential biases in the observed changes over time would be better understood by readers as a result.

**Interpretation of Socioeconomic Influences:** Although socioeconomic issues are mentioned in the article as having an impact on consanguinity practices, this topic might be further investigated. The analysis might be strengthened by a thorough examination of the ways in which acculturation, income, and educational attainment affect the prevalence of consanguineous marriage.

**Consideration of health outcomes: **A brief analysis or description of the health outcomes associated with consanguineous unions among the cohorts under study could give more context and highlight the public health concerns, even though the main focus is on prevalence.

**Community Perceptions:** Qualitative information about how the community views consanguinity would help the article better contextualize the trends. This could clarify whether societal factors, healthcare awareness, or other influences are the main drivers of these transitions and help explain the patterns that have been seen.

**Graphical Representation: **Trend graphs or demographic analyses of consanguinity trends are two examples of graphics that could better convey some of the findings. Both academic and non-academic readers would find the piece easier to read and more engaging as a result.

**Conclusion:**

 The work addresses a frequently disregarded facet of public health and community health dynamics, making a significant contribution to our understanding of consanguinity in the UK. Although the paper is valuable and well-researched, its depth and impact might be increased by including more in-depth socioeconomic analysis, health outcomes data, and community perspectives.

Is the work clearly and accurately presented and does it cite the current literature?

Yes

If applicable, is the statistical analysis and its interpretation appropriate?

Yes

Are all the source data underlying the results available to ensure full reproducibility?

Yes

Is the study design appropriate and is the work technically sound?

Yes

Are the conclusions drawn adequately supported by the results?

Yes

Are sufficient details of methods and analysis provided to allow replication by others?

Yes

Reviewer Expertise:

Human Genetics, Epidemiology, clinical and medical genetics, public health

I confirm that I have read this submission and believe that I have an appropriate level of expertise to confirm that it is of an acceptable scientific standard.

# Response

**Published:** 2024-11-26

**Authors:** Neil Small

**Affiliations:** Faculty of Health Studies, University of Bradford, Bradford, England, UK

We are very grateful for the supportive and insightful comments of our reviewer. We agree with the points that the paper’s depth and impact might be increased by including more in-depth socioeconomic analysis, health outcomes data, and community perspectives.  We have been working on allied and complimentary studies alongside the study our reviewer’s report relates to. When we posted this article, and when it was sent for review, two key pieces of work were still in development. These are now completed and are available to augment this article.

 Small N, Kelly B, Malawsky D S *et al.* Mortality, morbidity and educational outcomes in children of consanguineous parents in the Born in Bradford cohort [version 2; peer reviewed]. *Wellcome Open Res* 2024, 26 Sep 2024a, **9**:319 ( https://doi.org/10.12688/wellcomeopenres.22547.2)

 Small, N., Razaq, R et al. Changing patterns in marriage choice and related health risk in the Pakistani heritage community in Bradford UK: a qualitative study. Wellcome Open Research 22/11/24 9: 690 https://doi.org/10.12688//wellcomeopenres.23338.1 We have now cited them within this article.

**Areas reviewer 2 identified that would improve the article:**

***Clarification of Cohort Differences:*** *Despite the fact that two cohorts were examined, a more thorough comparison of the cohorts' sample attributes, recruiting strategies, and potential confounders will enhance the article. The comparability of the datasets and any potential biases in the observed changes over time would be better understood by readers as a result.*

***Interpretation of Socioeconomic Influences:** Although socioeconomic issues are mentioned in the article as having an impact on consanguinity practices, this topic might be further investigated. The analysis might be strengthened by a thorough examination of the ways in which acculturation, income, and educational attainment affect the prevalence of consanguineous marriage.*

 Re cohort differences and socioeconomic analysis – both our two cohort profiles – cited in the article (Wright et al 2013 and Dickerson et al 2022 ) and our two cohort protocols  (Raynor et al 2008 and Dickerson et al 2016)  – and material in our Extended data for this article and in Small et al 2024 give a lot of socioeconomic background and a lot of detail about the process of setting up and undertaking the cohort studies. We agree that further detailed analysis of specific factors would further strengthen our points. We hope to be able to consider such matters via exploration of specific research questions in future work. We also think deep analysis of differences between the cohorts would enhance future work on data from other areas of the cohort studies where comparison would be valuable. This article is, we hope, not the last in which we explore the benefits of cross-cohort comparison.

***Consideration of health outcomes:*** *A brief analysis or description of the health outcomes associated with consanguineous unions among the cohorts under study could give more context and highlight the public health concerns, even though the main focus is on prevalence.*

 We have looked at health outcomes – beyond recessive disorders -  using data that are available up to age 10 for all children in our cohort. They identify excess mortality and morbidity and also look at impact on educational attainment – see  Small et al 2024a. This reference  is now cited in the article.

***Community Perceptions:*** *Qualitative information about how the community views consanguinity would help the article better contextualize the trends. This could clarify whether societal factors, healthcare awareness, or other influences are the main drivers of these transitions and help explain the patterns that have been seen.*

 We have carried out a qualitative study with members of the  Bradford population who are of Pakistani heritage. They helped us explore changing attitudes to consanguinity and to the way health services respond to  it – see Small, Razaq et al 2024. This reference is now cited in the article.

***Graphical Representation:*** *Trend graphs or demographic analyses of consanguinity trends are two examples of graphics that could better convey some of the findings. Both academic and non-academic readers would find the piece easier to read and more engaging as a result.*

 We agree with this point and will consider these ways of presenting complex data in an accessible way in subsequent studies.

# Reviewer report: Reviewer response for version 1

**DOI:** 10.21956/wellcomeopenres.23363.r83070

**Published:** 2024-06-20

**Authors:** Parveen Ali

**Affiliations:** 1 University of Sheffield, Sheffield, UK

Thank you for submitting this manuscript which is written well and I only have a few questions/ comments/

 You mention in the abstract that ‘ *Research undertaken using the Born in Bradford cohort study identified consanguinity as a major risk factor for congenital anomalies and also reported longer term adverse health outcomes associated with consanguinity’.* It is a risk factor but don’t think it is a major risk factor

 There is not much difference in how scientific abstract and plain English Summary in reported. Plain English summary needs to be more accessible for lay audience.

 While consanguinity increases the risk of recessive disorders, at population level the risk is only 6% so not a huge high risk, however, the fact that it is concentrated in affected families and the conditions are severe, it is an important issue. Please clearly articulate this in your introduction to help reader understand context, else it reads as if every consanguineous couple is going to affected by recessive disorder which is not the case.

 What are the implications of the study, please state clearly in conclusion

Is the work clearly and accurately presented and does it cite the current literature?

Partly

If applicable, is the statistical analysis and its interpretation appropriate?

Yes

Are all the source data underlying the results available to ensure full reproducibility?

No source data required

Is the study design appropriate and is the work technically sound?

Yes

Are the conclusions drawn adequately supported by the results?

Yes

Are sufficient details of methods and analysis provided to allow replication by others?

Yes

Reviewer Expertise:

Inequalities in Health related to gender and ethnicity

I confirm that I have read this submission and believe that I have an appropriate level of expertise to confirm that it is of an acceptable scientific standard, however I have significant reservations, as outlined above.

# Response

**Published:** 2024-11-26

**Authors:** Neil Small

**Affiliations:** Faculty of Health Studies, University of Bradford, Bradford, England, UK

Parveen Ali – Reviewer 1.

***Comment One:*** *You mention in the abstract that ‘Research undertaken using the Born in Bradford cohort study identified consanguinity as a major risk factor for congenital anomalies and also reported longer term adverse health outcomes associated with consanguinity’. It is a risk factor but don’t think it is a major risk factor*

 We are grateful for this comment. We are aware that discussing risk involves complex considerations. Our research has found that consanguinity doubles risk of congenital anomalies and that no other factors (except age of mother – over 34 years) that have been considered in the literature (deprivation/co-morbidities etc) are supported by our data (Sheridan et al 2013). However, the considerable majority of children born to consanguineous parents will not have an anomaly (94%) and anomalies occur randomly in non-consanguineous children (at a rate of about 3%). We appreciate the reviewer’s observation that it needs to be clearer that recessive disorders are not evident in the offspring of a considerable majority of consanguineous parents and that this clarity also needs to be present in our Plain Language Summary.  We have added the following paragraph to our Introduction:   Congenital anomalies occur in all communities.  Rates are higher in children born to consanguineous parents although even here the considerable majority of children will not have an anomaly. In the BiB study about 3 in every 100 children born to non-consanguineous parents and about 6 in every 100 born to consanguineous partners were born with an anomaly. There are also other significant factors associated with increased presence of congenital anomalies. BiB reported an increase in risk of similar magnitude to consanguinity for non-consanguineous mothers of white British origin older than 34 years. Further, anomalies of different sorts and with risk factors other than consanguinity occur in all babies (Sheridan et al. 201 **3**). But even though congenital anomalies occur in only a small proportion of children, the consequences of being born with one can be severe (Small et al 2024a). These consequences include the death of the child or long term morbidity. Given that there are high numbers of consanguineous parents in Bradford, and despite most of their children not having a congenital anomaly, the numbers dying or living with the consequences of a congenital anomaly in Bradford are significant to the city and to its health services.

***Comment Two:*** *There is not much difference in how scientific abstract and plain English Summary in reported. Plain English summary needs to be more accessible for lay audience.*

*While consanguinity increases the risk of recessive disorders, at population level the risk is only 6% so not a huge high risk, however, the fact that it is concentrated in affected families and the conditions are severe, it is an important issue. Please clearly articulate this in your introduction to help reader understand context, else it reads as if every consanguineous couple is going to affected by recessive disorder which is not the case.*

 See responses to comment One re being clearer on the nature of risk. In addition to the changes made in responses  to comment one we have also rewritten our Plain Language Summary (pls) – our reviewer correctly points out that it closely replicated our Abstract – the new version does not do that, it makes a prominent point that a considerable majority of children born to consanguineous parents will not have an anomaly and it accords more closely with Wellcome Open guidance on requirements for the pls. New Plain language summary Born in Bradford  (BiB) studies health and well-being of children in this north of England city. Of interest has been the impact that parents who are blood relations (most often cousins) have on their children’s health. These unions increase the risk of one sort of genetic disorder, recessive genetic disorder. While the considerable majority of children born to cousins will not have these disorders the numbers who do are higher than in children of non-blood related parents. These disorders can have a severe impact. Cousin marriages are rare in the UK but are common in some communities, including the Pakistani heritage community in Bradford. Sixty-five percent of mothers recruited to BiB were of Pakistani heritage and 62% of these were married to cousins. BiB recruited women between 2007 and 2010. A second cohort of mothers were recruited between 2016 and 2019 (the Born in Bradford Better Start Study, BiBBS.) BiBBS also had a majority of women who were of Pakistani heritage. Over a nine year period there had been a reduction in the proportion of cousin marriages. Forty-six per-cent of Pakistani heritage women were related to the father of their baby in BiBBS. The reduction was most marked in women who were born in the UK, in those educated to A level or higher and in those under age 25. Comparing cohorts across time is a valuable way to examine social change. A reduction in cousin marriage is likely to reduce the number of recessive genetic disorders and be a positive contribution to the health of Bradford’s children. This study is from one city. Changes in Bradford may be attributable to   local factors;  partner choice may be different elsewhere.  But if they indicate a wider trend in Pakistani heritage communities then they could have widespread relevance.

***Comment three: **What are the implications of the study, please state clearly in conclusion*

 We have expanded our conclusions to try and make clearer our key implications. In addition,  implications are considered in our sections “Two cohort analysis” re methodological strengths of undertaking cohort studies with similar populations but with recruitment separated by a number of years. We also discuss how reasons for the behavioural changes we report can be explored qualitatively in our section on “Reasons for reductions in consanguinity” and we can now reference the qualitative study that complements this study and was not available when we first posted this paper – see Small, Razaq et al 2024. This modified paragraph now reads: Our data suggests some key areas to be explored but, in itself, does not explain why there has been a reduction. We have carried out qualitative research to explore reasons why members of the Pakistani heritage community in the city think rates of consanguinity might have changed over time. This qualitative research considers involvement in education, changes in migration patterns and differences in partner choices depending on age of women alongside other factors that were raised by our participants. We also looked at more distal factors including changes in economic wellbeing and shifts in the law and regulations shaping migration to the UK (Small, Razaq et al 2024). The main implication, described in our conclusion is that a drop in consanguinity in Bradford is likely to reduce the numbers of recessive disorders in children and also to reduce the higher mortality and morbidity we have shown to be associated with consanguinity, findings that have now been published and that  we can now cite in this study (Small et al 2024a).  We make the point that changes in Bradford may be attributable to local factors but if they indicate a wider trend in Pakistani heritage communities then they could have widespread relevance. Drops in rates of consanguinity are greatest in younger women, those under age 25 at the birth of their first child. Staying longer in education and being born in the UK are also linked to lower rates of consanguinity. This social change affects genetic health in individuals and, in communities where consanguinity is common, it has  wider planning and service provision implication. We have added the following to our conclusion: Knowledge of consanguinity prevalence and trends, and the detailed characteristics of who is in consanguineous unions, helps identify at-risk populations, and this can be used to enhance risk awareness and to help target genetic counselling. The identification of the reductions in consanguinity we report, in particular the considerable reductions in younger people, underscore a complex interplay of cultural, social, and demographic factors. It may be that we are seeing generational changes, and newly evolving societal norms. But these changes need to be monitored to see if they are indications of a lasting change and they need to be considered in other settings where consanguinity is common to see how widespread these reductions in consanguinity are.   These are the two additional articles that are linked with this article and were not available when this article was published and sent for review. These are now cited.

 .

 Small N, Kelly B, Malawsky D S *et al.* Mortality, morbidity and educational outcomes in children of consanguineous parents in the Born in Bradford cohort [version 2; peer reviewed]. *Wellcome Open Res* 2024, 26 Sep 2024, **9**:319 ( https://doi.org/10.12688/wellcomeopenres.22547.2) Small, N., Razaq, R et al 2024. Changing patterns in marriage choice and related health risk in the Pakistani heritage community in Bradford UK: a qualitative study. Wellcome Open Research 22/11/2024 9:690 https://doi.org/10.12688//wellcomeopenres.23338.1
